# Squamous cell carcinoma arising in mature ovarian teratoma: a rare clinical entity

**DOI:** 10.1093/omcr/omag003

**Published:** 2026-02-24

**Authors:** Yassine Hamdaoui, Achraf chetibi, Ines El achouri, Ayoub Kharkhach, Hassane Ait Ali, Tariq Bouhout, Badr Serji

**Affiliations:** Faculty of Medicine and Pharmacy, Mohammed First University, BP 724 Hay Al Quods, Oujda, Oriental Region, 60000, Morocco; Department of Surgical Oncology, Oncology Hospital, Mohammed VI University Hospital, BP 4806 Oujda Université 60049, Oriental Region, Morocco; Faculty of Medicine and Pharmacy, Mohammed First University, BP 724 Hay Al Quods, Oujda, Oriental Region, 60000, Morocco; Department of Surgical Oncology, Oncology Hospital, Mohammed VI University Hospital, BP 4806 Oujda Université 60049, Oriental Region, Morocco; Faculty of Medicine and Pharmacy, Mohammed First University, BP 724 Hay Al Quods, Oujda, Oriental Region, 60000, Morocco; Department of Surgical Oncology, Oncology Hospital, Mohammed VI University Hospital, BP 4806 Oujda Université 60049, Oriental Region, Morocco; Faculty of Medicine and Pharmacy, Mohammed First University, BP 724 Hay Al Quods, Oujda, Oriental Region, 60000, Morocco; Department of Surgical Oncology, Oncology Hospital, Mohammed VI University Hospital, BP 4806 Oujda Université 60049, Oriental Region, Morocco; Faculty of Medicine and Pharmacy, Mohammed First University, BP 724 Hay Al Quods, Oujda, Oriental Region, 60000, Morocco; Department of Surgical Oncology, Oncology Hospital, Mohammed VI University Hospital, BP 4806 Oujda Université 60049, Oriental Region, Morocco; Faculty of Medicine and Pharmacy, Mohammed First University, BP 724 Hay Al Quods, Oujda, Oriental Region, 60000, Morocco; Department of Surgical Oncology, Oncology Hospital, Mohammed VI University Hospital, BP 4806 Oujda Université 60049, Oriental Region, Morocco; Faculty of Medicine and Pharmacy, Mohammed First University, BP 724 Hay Al Quods, Oujda, Oriental Region, 60000, Morocco; Department of Surgical Oncology, Oncology Hospital, Mohammed VI University Hospital, BP 4806 Oujda Université 60049, Oriental Region, Morocco

**Keywords:** ovarian teratoma, squamous cell carcinoma, malignant transformation, peritoneal carcinomatosis, ovarian neoplasm

## Abstract

Squamous cell carcinoma (SCC) of the ovary is a rare malignancy, most commonly arising from malignant transformation within a mature cystic teratoma (MCT). The incidence of such transformation is estimated at around 2%. We report the case of a postmenopausal woman who initially underwent resection of a pelvic mass with right adnexectomy. Histopathological examination revealed squamous cell carcinoma arising in a mature ovarian teratoma. The case was subsequently discussed at a multidisciplinary tumor board, which recommended completion surgery. However, during the planned procedure, diffuse peritoneal carcinomatosis was discovered intraoperatively. The patient was therefore referred for neoadjuvant chemotherapy. This case highlights the diagnostic and therapeutic challenges of SCC arising in MCT, as well as its aggressive clinical course.

## Introduction

Mature cystic teratomas (MCTs), or dermoid cysts, are among the most frequent ovarian tumors, representing nearly one-third of all ovarian neoplasms and over half of benign cases. Although generally indolent, a small proportion—estimated between 0.2% and 1.4%—may undergo malignant transformation, most often into squamous cell carcinoma (SCC). This event is observed predominantly in women after menopause. The absence of specific symptoms, the poor sensitivity of tumor markers, and the limited value of imaging contribute to the diagnostic challenge, with confirmation usually obtained only after pathological examination. Prognosis is closely linked to disease stage: tumors confined to the ovary are associated with a more favorable outcome, whereas advanced disease is usually aggressive and carries a poor long-term survival.

Here, we describe the case of a postmenopausal patient in whom SCC developed within an ovarian teratoma, initially discovered as an adnexal mass. Surgical exploration and histology established the diagnosis, and subsequent management included multidisciplinary discussion and systemic therapy, reflecting the complexity of this condition.

## Case report

A 66-year-old postmenopausal woman, gravida 3 para 2 with a history of one abortion, presented with right lower abdominal pain lasting for approximately one month. Her medical history included type 2 diabetes mellitus, well controlled with oral medications, and hypertension managed with monotherapy. On physical examination, she was in good general condition, with no systemic symptoms such as weight loss or fever. There was no lymphadenopathy, breast abnormality, or neck swelling. Abdominal examination revealed a firm, mobile, non-tender abdominopelvic mass, which was confirmed on pelvic examination as a mobile adnexal mass [1, 2].

**Figure 1:**
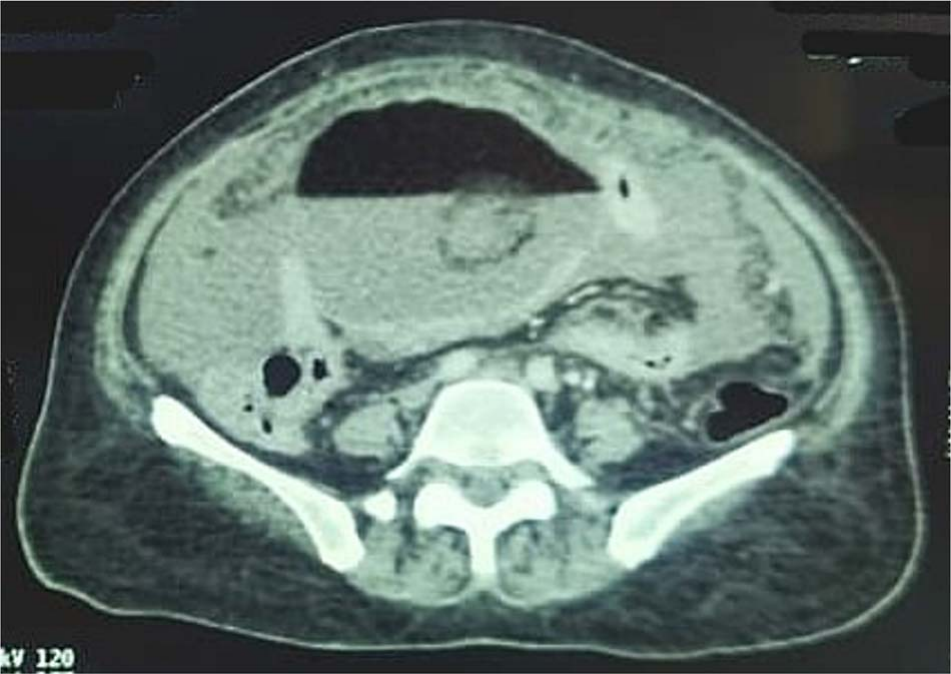
CT image showing a cystic ovarian mass with fatty and fluid components, calcification, and a Rokitansky nodule.

A CT scan of the abdomen demonstrated a large cystic abdominopelvic lesion containing fluid and fatty components, along with a calcification and a Rokitansky nodule, suggestive of a mature cystic teratoma. Minimal ascites was also noted (Fig. 1) [3].

MRI was not performed because the CT scan showed a typical appearance of a mature cystic teratoma (presence of fatty, calcified, and fluid components, as well as a Rokitansky nodule). The diagnosis was radiologically evident and did not warrant further imaging.

Tumor marker evaluation revealed an elevated CA-125 level of 176 U/mL [4].

**Figure 2:**
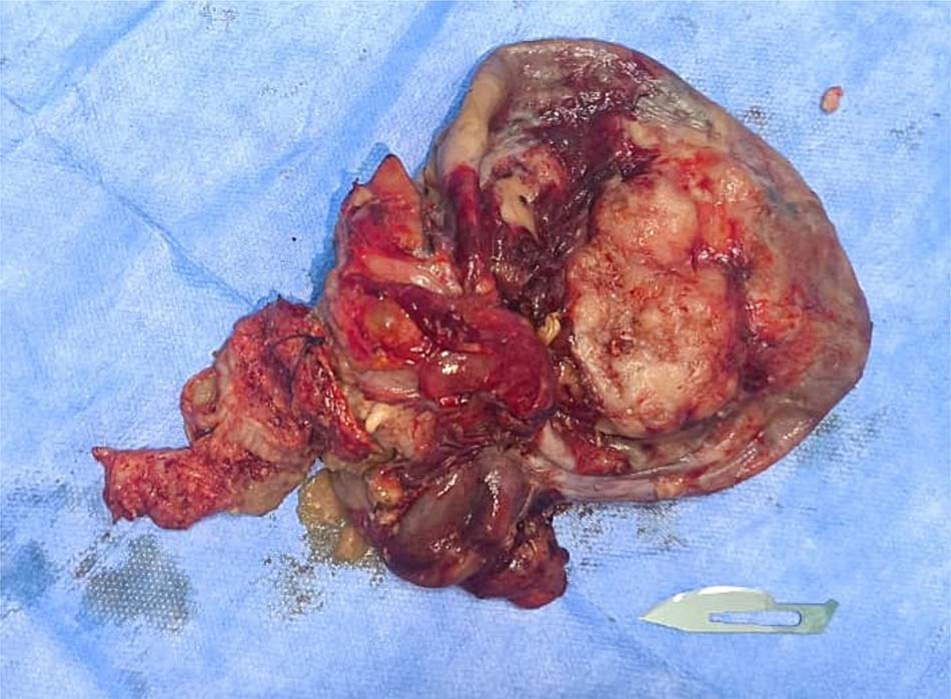
Intraoperative image of the resected pelvic mass.

**Figure 3:**
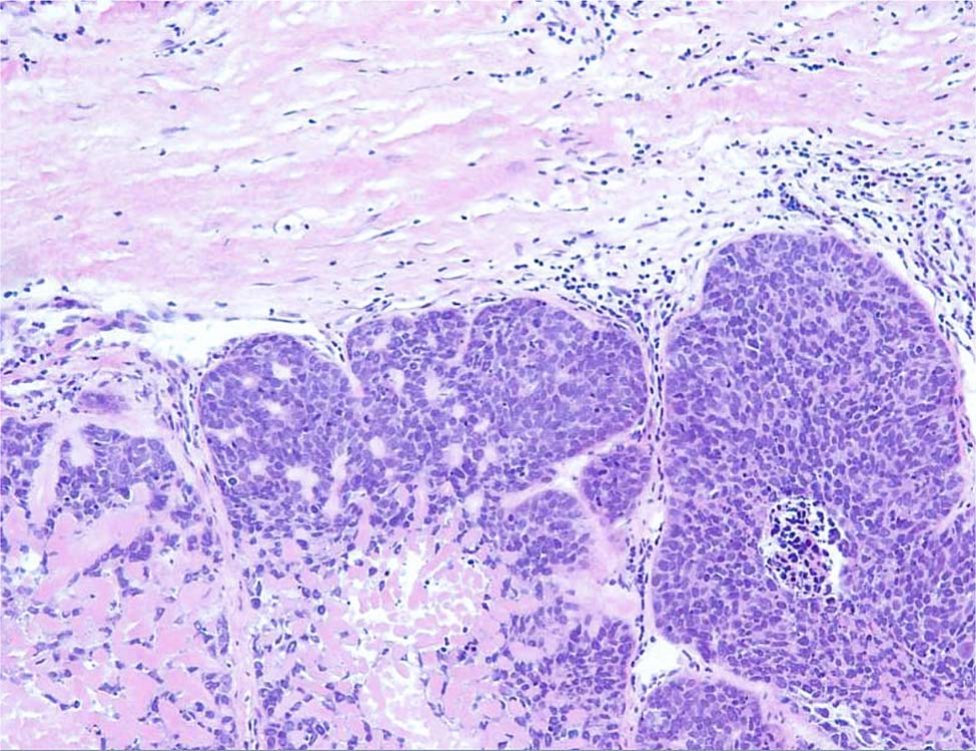
Histological section showing a well-differentiated squamous cell carcinoma arising within a mature ovarian teratoma (H&E stain).

The patient underwent surgical resection of the pelvic mass with right adnexectomy (Fig. 2). Histopathological examination confirmed a well-differentiated squamous cell carcinoma arising within a mature ovarian teratoma (TNM: T2aN0M0), showing nests of malignant squamous epithelial cells with keratinization and intercellular bridges (Fig. 3) [5, 6].

The case was reviewed by a multidisciplinary tumor board, which recommended completion surgery including total hysterectomy with bilateral adnexectomy for staging and cytoreduction. During re-exploration, diffuse peritoneal carcinomatosis was observed. Because of the extensive peritoneal spread, complete cytoreductive surgery (total hysterectomy, bilateral salpingo-oophorectomy, omentectomy, and lymph node dissection) was not feasible. The patient was therefore referred for neoadjuvant chemotherapy using the BEP regimen (Bleomycin, Etoposide, and Cisplatin), administered every three weeks for a total of six planned cycles [7, 8].

## Discussion

Squamous cell carcinoma (SCC) of the ovary is an uncommon malignancy, most frequently developing from a mature cystic teratoma (MCT), with reported incidence ranging from 1 to 2% [7]. Preoperative recognition is challenging because clinical symptoms and imaging findings are often nonspecific. MRI can suggest malignant transformation when solid areas or capsular invasion are present, but definitive diagnosis relies on histopathology. Tumor markers may provide diagnostic clues, with elevated CEA levels reported as more indicative of malignant transformation than CA-125 or CA 19–9. Although squamous cell carcinoma is the most frequent malignancy arising in mature cystic teratomas, other rare histologic transformation, such as adenocarcinoma or malignant melanoma, have also been described [8].

Prognosis remains poor, especially in advanced stages where complete cytoreduction is difficult. Risk factors associated with malignancy include postmenopausal status, larger tumor size (mean 15 cm versus 8 cm in benign cysts), presence of solid components, and invasion of adjacent organs or capsule [6]. In this case, the patient exhibited multiple risk factors, including postmenopausal age, elevated tumor markers, and a large, complex ovarian mass with solid areas.

Systematic reviews, including that by Hackethal et al., have shown that SCC in MCT predominantly occurs in women over 50, with tumors larger than 10 cm and elevated CA-125 levels [4]. Stage at diagnosis is the strongest prognostic factor: patients with stage I disease show markedly better survival compared to those with advanced-stage disease. Complete surgical resection followed by platinum-based chemotherapy has been associated with improved outcomes, whereas adjuvant radiotherapy has not demonstrated additional benefit [4, 9].

Other reports highlight the poor outcomes in stage III/IV disease. In a retrospective review of six such cases, median survival was only 12.5 months, although one patient achieved partial response with combined chemoradiotherapy, suggesting this approach may be considered in select situations [9]. Sporadic case reports from India have also described similar presentations, though some patients were younger, aged 37–40 years [10].

In the present case, histopathology confirmed SCC arising within an MCT. Initial management involved mass resection and right adnexectomy. Subsequent multidisciplinary discussion recommended completion surgery; however, intraoperative findings of diffuse peritoneal carcinomatosis placed the disease at an advanced stage, and the patient was referred for neoadjuvant chemotherapy. This case underscores the aggressive nature of ovarian SCC and highlights the critical role of multidisciplinary management in optimizing patient care.

## Conclusion

Squamous cell carcinoma arising in a mature cystic teratoma of the ovary is a rare but highly aggressive malignancy, often diagnosed at an advanced stage due to the absence of specific clinical or radiological features. Prognosis depends largely on stage at presentation, with poor survival in disseminated disease. Our case highlights the importance of considering malignant transformation in postmenopausal women presenting with large complex ovarian masses, the need for thorough histopathological evaluation, and the essential role of multidisciplinary management in optimizing treatment strategies.
